# Verifying OpenJDK’s LinkedList using KeY

**DOI:** 10.1007/978-3-030-45237-7_13

**Published:** 2020-03-13

**Authors:** Hans-Dieter A. Hiep, Olaf Maathuis, Jinting Bian, Frank S. de Boer, Marko van Eekelen, Stijn de Gouw

**Affiliations:** 8 Johannes Kepler University, Linz, Austria; 9 University of Birmingham, Birmingham, UK; 10 CWI, Science Park 123, 1098 XG Amsterdam, The Netherlands; 11 Open University, P.O. Box 2960, 6401 DL Heerlen, The Netherlands; 12 Achmea, P.O. Box 700, 7300 HC Apeldoorn, The Netherlands

**Keywords:** Java standard library, deductive verification, KeY, Java Modeling Language, case study, bug

## Abstract

As a particular case study of the formal verification of state-of-the-art, real software, we discuss the specification and verification of a corrected version of the implementation of a linked list as provided by the Java Collection framework.

## 1 Introduction

Software libraries are the building blocks of millions of programs, and they run on the devices of billions of users every day. Therefore, their correctness is of the utmost importance. The importance and potential of formal software verification as a means of rigorously validating state-of-the-art, real software and improving it, is convincingly illustrated by its application to TimSort, the default sorting library in many widely used programming languages, including Java and Python, and platforms like Android (see [7,9]): a crashing implementation bug was found.

The Java implementation of TimSort belongs to the Java Collection framework which provides implementations of basic data structures and is among the most widely used libraries. Nonetheless, over the years, 877 bugs in the Collections Framework have been reported in the official OpenJDK bug tracker.

Due to the intrinsic complexity of modern software, the possibility of interventions by a human verifier is indispensable for proving correctness. This holds in particular for the Java Collection library, where programs are expected to behave correctly for inputs of arbitrary size. As a particular case study, we discuss the formal verification of a corrected version of the implementation of a linked list as specified by the class `LinkedList` of the Java Collection framework in Java 8. Apart from the fact that the data structure of a linked list is one of the basic structures for storing and maintaining unbounded data, this is an interesting case study because it provides further evidence that formal verification of real software can lead to major improvements and correctness guarantees.

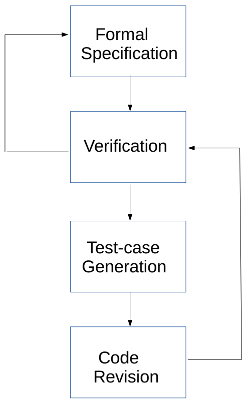

Fig. 1: Workflow

We follow the general workflow underlying the TimSort case as depicted in Fig. 1. The workflow starts with a formalisation of the informal documentation of the Java code in the Java Modeling Language [10,16]. This formalisation goes hand in hand with the formal verification: failed verification attempts can provide information about further refinements of the specs. A failed verification attempt may also indicate an error in the code, and can as such be used for the generation of test cases to detect the error at run-time.

`LinkedList` is the only `List` implementation in the Collection Framework that allows collections of unbounded size. During verification we found out that the Java linked list implementation does not correctly take into account the Java integer overflow semantics. It is exactly for large lists ($\geq 2^{31}$ items), that the implementation breaks. This basic observation gave rise to a number of test cases which show that Java's `LinkedList` class breaks 22 methods out of a total of 25 methods of the `List`![4]

On the basis of these test cases we propose in Sect. 2 a code revision of the Java linked list implementation, and formally specify and verify its correctness in Sect. 3 with respect to the Java integer overflow semantics. Section 4 discusses the main challenges posed by this case study and related work.

This case study has been carried out using the state-of-the-art KeY theorem prover [3], because it formalizes the integer overflow semantics of Java and it allows to directly "load" Java programs. An archive of proof files and the KeY version used in this study is available on-line in the Zenodo repository [2].

## 2 `LinkedList` in OpenJDK

`LinkedList` was introduced in Java version 1.2 as part of Java's Collection Framework in 1998. The `LinkedList` class is part of the type hierarchy of this framework: `LinkedList` implements the `List` interface, and also supports all general `Collection` methods as well as the methods from the `Queue` and `Deque` interfaces. The `List` interface provides positional access to the elements of the list, where each element is indexed by Java's primitive `int` type.

The structure of the `LinkedList` class is shown in Listing 1. This class has three attributes: a `size` field, which stores the number of elements in the list, and two fields that store a reference to the `first` and `last` node. Internally, it uses the private static nested `Node` class to represent the items in the list. A static nested private class behaves like a top-level class, except that it is not visible outside the enclosing class (`LinkedList`, in this case). Nodes are doubly linked; each node is connected to the preceding (field `prev`) and succeeding node

[4] We filed a bug report to Oracle's security team. Once the report is made public by the Java maintainers, we will add the URL as metadata to our repository [2].

```
public class LinkedList<E>
      extends AbstractSequentialList<E>
      implements List<E>, Deque<E>, ... {
  transient int size = 0;
  transient Node<E> first;
  transient Node<E> last;
  private static class Node<E> {
    E item;
    Node<E> next;
    Node<E> prev;
    Node(Node<E> p, E i, Node<E> n) ...
  }
  ...
}
```

```
public boolean add(E e) {
  linkLast(e);
  return true;
}
void linkLast(E e) {
  final Node<E> l = last;
  final Node<E> newNode =
      new Node<>(l, e, null);
  last = newNode;
  if (l == null) first = newNode;
  else l.next = newNode;
  size++;
  modCount++;
}
```

Listing 1: The `LinkedList` class defines a doubly-linked list data structure.

```
public int indexOf(Object o) {
    int index = 0;
    if (o == null) {
        for (Node<E> x = first; x != null; x = x.next) {
            if (x.item == null)
                return index;
            index++;
        }
    } else {
        for (Node<E> x = first; x != null; x = x.next) {
            if (o.equals(x.item))
                return index;
            index++;
        }
    }
    return -1;
}
```

Listing 2: The `indexOf` method searches for an element from the first node on.

(field `next`). These fields contain `null` in case no preceding or succeeding node exists. The data itself is contained in the `item` field of a node.

`LinkedList` contains 57 methods. Due to space limitations, we now focus on three characteristic methods: see Listing 1 and Listing 2. Method `add(E)` calls method `linkLast(E)`, which creates a new `Node` object to store the new item and adds the new node to the end of the list. Finally the new size is determined by unconditionally incrementing the value of the `size` field, which has type `int`. Method `indexOf(Object)` returns the position (of type `int`) of the first occurrence of the specified element in the list, or $-1$ if it's not present.

Each linked list consists of a sequence of nodes. Sequences are finite, indexing of sequences starts at zero, and we write $\sigma[i]$ to mean the $i$th element of some sequence $\sigma$. A *chain* is a sequence $\sigma$ of nodes of length $n > 0$ such that: the `prev` reference of the first node $\sigma[0]$ is `null`, the `next` reference of the last node $\sigma[n-1]$ is `null`, the `prev` reference of node $\sigma[i]$ is node $\sigma[i-1]$ for every index $0 < i < n$, and the `next` reference of node $\sigma[i]$ is node $\sigma[i+1]$ for every index $0 \leq i < n-1$. The `first` and `last` references of a linked list are either both `null` to represent the *empty* linked list, or there is some chain $\sigma$ between the `first` and `last` node, viz. $\sigma[0] =$ `first` and $\sigma[n-1] =$ `last`. Figure 2 shows example instances. Also see standard literature such as Knuth's [15, Section 2.2.5].

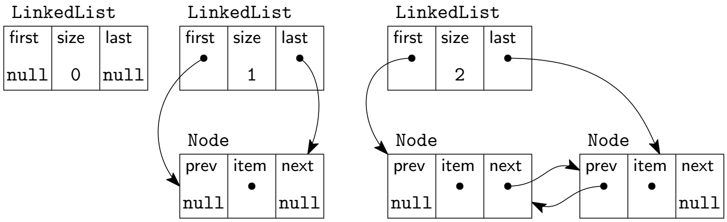

Fig. 2: Three example linked lists: empty, with a chain of one node, and with a chain of two nodes. Items themselves are not shown.

We make a distinction between the *actual* size of a linked list and its *cached* size. In principle, the size of a linked list can be computed by walking through the chain from the `first` to the `last` node, following the `next` reference, and counting the number of nodes. For performance reasons, the Java implementation also maintains a cached size. The cached size is stored in the linked list instance.

Two basic properties of doubly-linked lists are *acyclicity* and *unique first and last nodes*. Acyclicity is the statement that for any indices $0 \leq i < j < n$ the nodes $\sigma[i]$ and $\sigma[j]$ are different. First and last nodes are unique: for any index $i$ such that $\sigma[i]$ is a node, the `next` of $\sigma[i]$ is `null` if and only if $i = n - 1$, and `prev` of $\sigma[i]$ is `null` if and only if $i = 0$. Each item is stored in a separate node, and the same item may be stored in different nodes when duplicate items are present in the list.

## 2.1 Integer overflow bug

The size of a linked list is encoded by a signed 32-bit integer (Java's primitive `int` type) that has a two's complement binary representation where the most significant bit is a sign bit. The values of `int` are bounded and between $-2^{31}$ (`Integer.MIN_VALUE`) and $2^{31}-1$ (`Integer.MAX_VALUE`), inclusive. Adding one to the maximum value, $2^{31} - 1$, results in the minimum value, $-2^{31}$: the carry of addition is stored in the sign bit, thereby changing the sign.

Since the linked list implementation maintains one node for each element, its size is implicitly bounded by the number of node instances that can be created. Until 2002, the JVM was limited to a 32-bit address space, imposing a limit of 4 gigabytes (GiB) of memory. In practice this is insufficient to create $2^{31}$ node instances. Since 2002, a 64-bit JVM is available allowing much larger amounts of addressable memory. Depending on the available memory, in principle it is now possible to create $2^{31}$ or more node instances. In practice such lists can be constructed today on systems with 64 gigabytes of memory, e.g., by repeatedly adding elements. However, for such large lists, at least 20 methods break, caused by signed integer overflow. For example, several methods crash with a run-time exception or exhibit unexpected behavior!

Integer overflow bugs are a common attack vector for security vulnerabilities: even if the overflow bug may seem benign, its presence may serve as a small step in a larger attack. Integer overflow bugs can be exploited more easily on large memory machines used for 'big data' applications. Already, real-world attacks involve Java arrays with approximately $2^{32}/5$ elements [11, Section 3.2].

The `Collection` interface allows for collections with over `Integer.MAX_-VALUE` elements. For example, its documentation (Javadoc) explicitly states the behavior of the `size()` method: 'Returns the number of elements in this collection. If this collection contains more than `Integer.MAX_VALUE` elements, returns `Integer.MAX_VALUE`'. The special case ('more than …') for large collections is necessary because `size()` returns a value of type `int`.

When `add(E)` is called and unconditionally increments the `size` field, an overflow happens after adding $2^{31}$ elements, resulting in a negative `size` value. In fact, as the Javadoc of the `List` interface describes, this interface is based on integer indices of elements: 'The user can access elements by their integer index (position in the list), …'. For elements beyond `Integer.MAX_VALUE`, it is very unclear what integer index should be used. Since there are only $2^{32}$ different integer values, at most $2^{32}$ node instances can be associated with an unique index. For larger lists, elements cannot be uniquely addressed anymore using an integer index. In essence, as we shall see in more detail below, the bounded nature of the 32-bit integer indices implies that the design of the `List` interfaces breaks down for large lists on 64-bit architectures. The above observations have many ramifications: it can be shown that 22 of 25 methods in the `List` interface are broken. Remarkably, the actual size of the linked list remains correct as the chain is still in place: most methods of the `Queue` interface still work.

## 2.2 Reproduction

We have run a number of test cases to show the presence of bugs caused by the integer overflow. The running Java version was Oracle's JDK8 (build 1.8.0 201-b09) that has the same `LinkedList` implementation as in OpenJDK8. Before running a test case, we set up an empty linked list instance. Below, we give an high-level overview of the test cases. Each test case uses `letSizeOverflow()` or `addElementsUntilSizeIs0()`: these repeatedly call the method `add()` to fill the linked list with `null` elements, and the latter method also adds a last element (`"this is the last element"`) causing `size` to be 0 again.

1. Directly after `size` overflows, the `size()` methods returns a negative value, violating what the corresponding Javadoc stipulates: its value should remain `Integer.MAX_VALUE` $= 2^{31} - 1$.

```
   letSizeOverflow();
   System.out.println("linkedList.size() = " + linkedList.size() + ", actual: " + count);
   // linkedList.size() = -2147483648, actual: 2147483648
   ```

Clearly this behavior is in contradiction with the documentation. The actual number of elements is determined by having a field `count` (of type `long`) that is incremented each time the method `add()` is called.
2. The query method `get(int)` returns the element at the specified position in the list. It throws an `IndexOutOfBoundsException` exception when `size` is negative. From the informal specification, it is unclear what indices should be associated with elements beyond `Integer.MAX_VALUE`.

```
letSizeOverflow();
System.out.println(linkedList.get(0));
// Exception in thread "main" IndexOutOfBoundsException: Index: 0, Size: -2147483648
//        at java.util.LinkedList.checkElementIndex(LinkedList.java:555) ...
```

3. The method `toArray()` returns an array containing all of the elements in this list in proper sequence (from first to last element). When `size` is negative, this method throws a `NegativeArraySizeException` exception. Furthermore, since the array size is bounded by $2^{31} - 1$ elements[5], the contract of `toArray()` is unsatisfiable for lists larger than this. The method `Collections.sort(List<T>)` sorts the specified list into ascending order, according to the natural ordering of its elements. This method calls `toArray()`, and therefore also throws a `NegativeArraySizeException`.

```
letSizeOverflow();
Collections.sort(linkedList);
// Exception in thread "main" NegativeArraySizeException
//        at java.util.LinkedList.toArray(LinkedList.java:1050)...
```

4. Method `indexOf(Object o)` returns the index of the first occurrence of the specified element in this list, or $-1$ if this list does not contain the element. However due to the overflow, it is possible to have an element in the list associated to index $-1$, which breaks the contract of this method.

```
addElementsUntilSizeIs0();
String last;
System.out.println("linkedList.getLast() = " + (last = linkedList.getLast()));
// linkedList.getLast() = This is the last element
System.out.println("linkedList.indexOf(" + last + ") = " + linkedList.indexOf(last));
// linkedList.indexOf(This is the last element) = -1
```

5. Method `contains(Object o)` returns true if this list contains the specified element. If an element is associated with index $-1$, it will indicate wrongly that this particular element is not present in the list.

```
addElementsUntilSizeIs0();
String last;
System.out.println("linkedList.getLast() = " + (last = linkedList.getLast()));
// linkedList.getLast() = This is the last element
System.out.println("linkedList.contains(" + last + ") = " linkedList.contains(last));
// linkedList.contains(This is the last element) = false
```

Specifically, method `letSizeOverflow()` adds $2^{31}$ elements that causes the overflow of `size`. Method `addElementsUntilSizeIs0()` first adds $2^{32} - 1$ elements: the value of `size` is then $-1$. Then, it adds the last element, and `size` is 0 again. All elements added are `null`, except for the last element. For test cases 4 and 5, we deliberately misuse the overflow bug to associate an element with index $-1$. This means that method `indexOf(Object)` for this element returns $-1$, which according to the documentation means that the element is not present. For test cases 1, 2 and 3 we needed 65 gigabytes of memory for the JRE on a VM with 67 gigabytes of memory. For test cases 4 and 5 we needed 167 gigabytes of memory for the JRE on a VM with 172 gigabytes of memory. All test cases were carried out on a machine in a private cloud (SURFsara), which provides instances that satisfy these system requirements.

[5] In practice, the maximum array length turns out to be $2^{31} - 5$, as some bytes are reserved for object headers, but this may vary between Java versions [11,14].

### 2.3 Mitigation

There are multiple directions for mitigating the overflow bug: *do not fix*, *fail fast*, *long size field* and *long or `BigInteger` indices*. Due to lack of space, we describe only the *fail fast* solution. This solution stays reasonably close to the original implementation of `LinkedList` and does not leave any behavior unspecified.

In the *fail fast* solution, we ensure that the overflow of `size` may never occur. Whenever elements would be added that cause the size field to overflow, the operation throws an exception and leaves the list unchanged. As the exception is triggered right before the overflow would otherwise occur, the value of `size` is guaranteed to be bounded by `Integer.MAX_VALUE`, i.e. it never becomes negative.

This solution requires a slight adaptation of the implementation: for methods that increase the `size` field, only one additional check has to be performed before a `LinkedList` instance is modified. This checks whether the result of the method causes an overflow of the `size` field. Under this condition, an `IllegalStateException` is thrown. Thus, only in states where `size` is less than `Integer.MAX_VALUE`, it is acceptable to add a single element to the list.

We shall work in a separate class called `BoundedLinkedList`: this is the improved version that does not allow more than $2^{31}-1$ elements. Compared to the original `LinkedList`, two methods are added, `isMaxSize()` and `checkSize()`:

```
private boolean isMaxSize() {
  return size == Integer.MAX_VALUE;
}
private void checkSize() {
  if (isMaxSize())
    throw new IllegalStateException("Not enough space");
}
```

These methods implement an overflow check. The latter method is called before any modification occurs that increases the size by one: this ensures that `size` never overflows. Some methods now differ when compared to the original `LinkedList`, as they involve an invocation of the `checkSize()` method.

## 3 Specification and verification of `BoundedLinkedList`

The aim of our specification and verification effort is to verify formalizations of the given Javadoc specifications (stated in natural language) of the `LinkedList`. This includes establishing absence of overflow errors. Moreover, we restrict our attention only to the revised `BoundedLinkedList` and not to the rest of the Collection Framework or Java classes: methods that involve parameters with interface types, Java serialization or Java reflection are considered out of scope.

`(Bounded)LinkedList` inherits from `AbstractSequentialList`, but we consider its inherited methods out of scope. These methods operate on other collections such as `removeAll` or `containsAll`, and methods that have other classes as return type such as `iterator`. However, these methods call methods overridden by `(Bounded)LinkedList`, and can not cause an overflow by themselves.

We have made use of KeY's stub generator to generate dummy contracts for other classes that `BoundedLinkedList` depends on, such as for the inherited interfaces and abstract super classes: these contracts conservatively specify that every method may arbitrarily change the heap. The stub generator moreover deals with generics by erasing the generic type parameters. For exceptions we modify their stub contract to assume that their constructors are *pure*, viz. leaving existing objects on the heap unchanged. An important stub contract is the equality method of the absolute super class `Object`, which we have adapted: we assume every object has a *side-effect free*, *terminating* and *deterministic* implementation of its equality method[6]:

```
public class Object {
    /*@ public normal_behavior
      @   requires true;
      @   ensures \result == self.equals(param0);
      @*/
    public /*@ helper strictly_pure @*/ boolean
      equals(/*@ nullable */ Object param0);
    ...
}
```

### 3.1 Specification

Following our workflow, we have iterated a number of times before the specifications we present here were obtained. This is a costly procedure, as revising some specifications requires redoing most verification effort. Until sufficient information is present in the specification, proving for example termination of a method is difficult or even impossible: from stuck verification attempts, and an intuitive idea of why a proof is stuck, the specification is revised.

*Ghost fields.* We use JML's ghost fields: these are logical fields that for each object gets a value assigned in a heap. The value of these fields are conceptual, i.e. only used for specification and verification purposes. During run-time, this field is not present and cannot affect the course of execution. Our improved class is annotated with two ghost fields: `nodeList` and `nodeIndex`.

The type of the `nodeList` ghost field is an abstract data type of sequences, a KeY built-in. This type has standard constructors and operations that can be used in contracts and in JML set annotations. A sequence has a length, which is finite but unbounded. The type of a sequence's length is `\bigint`. In KeY a sequence is unityped: all its elements are of the *any* sort, which can be any Java object reference or primitive, or built-in abstract data type. One needs to apply appropriate casts and track type information for a sequence of elements in order to cast elements of the *any* sort to any of its subsorts.

The `nodeIndex` ghost field is used as a ghost parameter with unbounded but finite integers as type. This ghost parameter is only used for specifying the behavior of the methods `unlink(Node)` and `linkBefore(Object, Node)`. The ghost parameter tracks at which index the `Node` argument is present in the `nodeList`. This information is implicit and not needed at run-time.

[6] In reality, there are Java classes for which equality is not terminating. A nice example is `LinkedList` itself, where adding a list to itself leads to a `StackOverflowError` when testing equality with a similar instance. We consider the issue out of scope of this study as this behavior is explicitly described by the Javadoc.

*Class invariant.* The ghost field `nodeList` is used in the class invariant of our improved implementation, see below. We relate the fields `first` and `last` that hold a reference to a `Node` instance, and the chain between `first` and `last`, to the contents of the sequence in the ghost field `nodeList`. This allows us to express properties in terms of `nodeList`, where they reflect properties about the chain on the heap. One may compare this invariant with the description of chains as given in Sect. 2.

```
1  //@ private ghost \seq nodeList;
2  //@ private ghost \bigint nodeIndex;
3  /*@ invariant
4    @   nodeList.length == size &&
5    @   nodeList.length <= Integer.MAX_VALUE &&
6    @   (\forall \bigint i; 0 <= i < nodeList.length;
7    @       nodeList[i] instanceof Node) &&
8    @   ((nodeList == \seq_empty && first == null && last == null)
9    @    || (nodeList != \seq_empty && first != null &&
10   @         first.prev == null && last != null &&
11   @         last.next == null && first == (Node)nodeList[0] &&
12   @         last == (Node)nodeList[nodeList.length-1])) &&
13   @   (\forall \bigint i; 0 < i < nodeList.length;
14   @       ((Node)nodeList[i]).prev == (Node)nodeList[i-1]) &&
15   @   (\forall \bigint i; 0 <= i < nodeList.length-1;
16   @       ((Node)nodeList[i]).next == (Node)nodeList[i+1]);
17   @*/
```

The actual size of a linked list is the length of the ghost field `nodeList`, whereas the cached size is stored in a 32-bit signed integer field `size`. On line 4, the invariant expresses that these two must be equal. Since the length of a sequence (and thus `nodeList`) is never negative, this implies that the size field never overflows. On line 5, this is made explicit: the real size of a linked list is bounded by `Integer.MAX_VALUE`. Line 5 is redundant as it follows from line 4, since a 32-bit integer never has a value larger than this maximum value. The condition on lines 6–7 requires that every node in `nodeList` is an instance of `Node` which implies it is non-`null`.

A linked list is either empty or non-empty. On line 8, if the linked list is empty, it is specified that `first` and `last` must be `null` references. On lines 9–12, if the linked list is non-empty, it is specified that `first` and `last` are non-`null` and moreover that the `prev` field of the first `Node` and the `next` field of the last `Node` are `null`. The `nodeList` must have as first element the node pointed to by `first`, and `last` as last element. In any case, but vacuously true if the linked list is empty, the `nodeList` forms a chain of nodes: lines 13–16 describe that, for every node at index $0 < i <$ `size`, the `prev` field must point to its predecessor, and similar for successor nodes.

We note three interesting properties that are implied by the above invariant: acyclicity, unique first and unique last node. These properties can be expressed as JML formulas as follows:

```
(\forall \bigint i; 0 <= i < nodeList.length - 1;
    (\forall \bigint j; i < j < nodeList.length;
        nodeList[i] != nodeList[j])) &&
(\forall \bigint i; 0 <= i < nodeList.length;
    nodeList[i].next == null <==> i = nodeList.length - 1) &&
(\forall \bigint i; 0 <= i < nodeList.length;
    nodeList[i].prev == null <==> i = 0)
```

These properties are not literally part of our invariant, but their validity is proven interactively in KeY as a consequence of the invariant. Otherwise, we would need to reestablish also these properties each time we show the invariant holds.

*Methods.* All methods within scope are given a JML contract that specify its normal behavior and its exceptional behavior. As an example contract, consider the `lastIndexOf(Object)` method in Listing 3: it searches through the chain of nodes until it finds a node with an item equal to the argument. This method is interesting due to a potential overflow of the resulting index. `BoundedLinkedList` together with all method specifications are available on-line [2].

### 3.2 Verification

We start by giving a general strategy we apply to verify proof obligations. We also describe in more detail how to produce a single proof, in this case `lastIndexOf(Object)`. This gives a general feel how proving in KeY works. This method is neither trivial, nor very complicated to verify. In this manner, we have produced proofs for each method contract that we have specified.

*Overview of verification steps.* When verifying a method, we first instruct KeY to perform symbolic execution. Symbolic execution is implemented by a number of proof rules that transform modal operators on program fragments in `JavaDL`. During symbolic execution, the goal sequent is automatically simplified, potentially leading to branches. Since our class invariant contains a disjunction (either the list is empty or not), we do not want these cases to be split early in the symbolic execution. Thus we instruct KeY to delay unfolding the class invariant. When symbolic execution is finished, goals may still contain updated heap expressions that must be simplified further. After this has been done, one can compare the open goals to the method body and its annotations, and see whether the open goals in KeY look familiar and check whether they are true.

In the remaining part of the proof the user must find an appropriate mix between interactive and automatic steps. If a sequent is provable, there may be multiple ways to construct a closed proof tree. At (almost) every step the user has a choice between applying steps manually or automatically. It requires some experience in choosing which rules to apply manually: clever rule application decreases the size of the proof tree. Certain rules are never applied automatically, such as the cut rule. The cut rule splits a proof tree into two parts by introducing a detour, but significantly reduces the size of a proof and thus the effort required to produce it. For example, the acylicity property can be introduced using cut.

*Verification example.* The method `lastIndexOf` has two contracts: one involves a `null` argument, and another involves a non-`null` argument. Both proofs are similar. Moreover, the proof for `indexOf(...)` is similar but involves the `next` reference instead of the `prev` reference. This contract is interesting, since proving its correctness shows the absence of the overflow of the `index` variable.

**Proposition.** `lastIndexOf(Object)` *as specified in Listing 3 is correct.*

*Proof.* Set strategy to default strategy, and set max. rules to 5,000, class axiom delayed. Finish symbolic execution on the main goal. Set strategy to 1,000 rules

```
/*@
  @ also
  @ ...
  @ public normal_behavior
  @   requires
  @     o != null;
  @   ensures
  @     \result >= -1 && \result < nodeList.length;
  @   ensures
  @     \result == -1 ==>
  @       (\forall \bigint i; 0 <= i < nodeList.length;
  @         !o.equals(((Node)nodeList[i]).item));
  @   ensures
  @     \result >= 0 ==>
  @       (\forall \bigint i; \result < i < nodeList.length;
  @         !o.equals(((Node)nodeList[i]).item)) &&
  @       o.equals(((Node)nodeList[\result]).item);
  @*/
public /*@ strictly_pure @*/ int
lastIndexOf(/*@ nullable @*/ Object o) {
    int index = size;
    if (o == null) {
        ...
    } else {
        /*@
          @ maintaining
          @   (\forall \bigint i; index <= i < nodeList.length;
          @       !o.equals(((Node)nodeList[i]).item));
          @ maintaining
          @   0 <= index && index <= nodeList.length;
          @ maintaining
          @   0 < index && index <= nodeList.length ==>
          @     x == (Node)nodeList[index - 1];
          @ maintaining
          @   index == 0 <==> x == null;
          @ decreasing
          @   index;
          @ assignable
          @   \strictly_nothing;
          @*/
        for (Node x = last; x != null; x = x.prev) {
            index--;
            if (o.equals(x.item))
                return index;
        }
    }
    return -1;
}
```

Listing 3: Method `lastIndexOf(Object)` annotated with JML. Searches the list from last to first for an element. Returns $-1$ if this element is not present in the list; otherwise returns the index of the node that was equal to the argument. Only the contract and branch in which the argument is non-`null` is shown due to space restrictions. Methods such as `indexOf`, `removeFirstOccurrence` and `removeLastOccurrence` are very similar.

and select DefOps arithmetical rules. Close all provable goals under the root. One goal remains. Perform update simplification macro on the whole sequent, perform propositional with split macro on the sequent, and close provable goals on the root of the proof tree. There is a remaining case:

– Case $\mathit{index} - 1 = 0 \leftrightarrow x.\mathtt{prev} = \mathtt{null}$: split the equivalence. First case, suppose $\mathit{index} - 1 = 0$, then $x = \mathit{self}.\mathtt{nodeList}[0] = \mathit{self}.\mathtt{first}$ and $\mathit{self}.\mathtt{first}.\mathtt{prev} = \mathtt{null}$: solvable through unfolding the invariant and equational rewriting. Now, second case, suppose $x.\mathtt{prev} = \mathtt{null}$. Then, either $\mathit{index} = 1$ or $\mathit{index} > 1$ (from splitting $\mathit{index} \geq 1$). The first of which is trivial (close provable goal), and the second one requires instantiating quantified statements from the invariant, leading to a contradiction. Since we have supposed $x.\mathtt{prev} = \mathtt{null}$, but $x = \mathit{self}.\mathtt{nodeList}[\mathit{index} - 1]$ and $\mathit{self}.\mathtt{nodeList}[\mathit{index} - 1].\mathit{prev} = \mathit{self}.\mathtt{nodeList}[\mathit{index} - 2]$ and $\mathit{self}.\mathtt{nodeList}[\mathit{index} - 2] \neq \mathtt{null}$. □

*Interesting verification conditions.* The acyclicity property is used to close verification conditions that arise as a result of potential aliasing of node instances: it is used as a separation lemma. For example, after a method that changed the `next` field of an existing node, we want to reestablish that all nodes remain reachable from the `first` through `next` fields (i.e., "connectedness"): one proves that the update of `next` only affects a single node, and does not introduce a cycle. We prove this by using the fact that two nodes instances are different if they have a different index in `nodeList`, which follows from acyclicity. Below, we sketch an argument why the acyclicity property follows from the invariant. We have a video in which we show how the argument in KeY goes, see [1, 0:55–11:30].

**Proposition.** *Acyclicity follows from the linked list invariant.*

*Proof.* By contradiction: suppose a linked list of size $n > 1$ is not acyclic. Then there are two indices, $0 \leq i < j < n$, such that the nodes at index $i$ and $j$ are equal. Then it must hold that for all $j \leq k < n$, the node at $k$ is equal to the node at $k - (j - i)$. This follows from induction. Base case: if $k = j$, then node $j$ and node $j - (j - i) = i$ are equal by assumption. Induction step: suppose node at $k$ is equal to node at $k - (j - i)$, then if $k + 1 < n$ it also holds that node $k + 1$ equals node $k + 1 - (j - i)$: this follows from the fact that node $k + 1$ and $k + 1 - (j - i)$ are both the `next` of node $k < n - 1$ and node $k - (j - i)$. Since the latter are equal, the former must be equal too. Now, for all $j \leq k < n$, node $k$ equals node $k - (j - i)$ in particular holds when $k = n - 1$. However, by the property that only the last node has a `null` value for `next`, and a non-last node has a non-`null` value for its `next` field, we derive a contradiction: if nodes $k$ and $k - (j - i)$ are equal then all their fields must also have equal values, but node $k$ has a `null` and node $k - (j - i)$ has a non-`null` next field! □

*Summary of verification effort.* The total effort of our case study was about 7 man months. The largest part of this effort is finding the right specification. KeY supports various ways to specify Java code: model fields/methods, pure methods, and ghost variables. For example, using pure methods, contracts are specified by expressing the content of the list before/after the method using the pure method get(i), which returns the item at index $i$. This led to rather complex proofs: essentially it led to reasoning in terms of relational properties on programs (i.e. `get(i)` before vs get(i) after the method under consideration). After 2.5 man months of writing partial specifications and partial proofs in these different formalisms, we decided to go with ghost variables as this was the only formalism in which we succeeded to prove non-trivial methods.

It then took $\approx$ 4 man months of iterating in our workflow through (failed) partial proof attempts and refining the specs until they were sufficiently complete. In particular, changes to the class invariant were "costly", as this typically caused proofs of all the methods to break (one must prove that all methods preserve the class invariant). The possibility to interact with the prover was crucial to pinpoint the cause of a failed verification attempt, and we used this feature of KeY extensively to find the right changes/additions to the specifications.

After the introduction of the field `nodeList`, several methods could be proved very easily, with a very low number of interactive steps or even automatically. Methods `unlink(Node)` and `linkBefore(Object, Node)` could not be proven without knowing the position of the node argument. We introduced a new ghost field, `nodeIndex`, that acts like a ghost parameter. Luckily, this did not affect the class invariant, and existing proofs that did not make use of the new ghost field were unaffected.

Once the specifications are (sufficiently) complete, we estimate that it only took approximately 1 or 1.5 man weeks to prove all methods. This can be reduced further if informal proof descriptions are given. Moreover, we have recorded a video of a 30 minute proof session where the method `unlinkLast` is proven correct with respect to its contract [1].

*Proof statistics.* The below table summarizes the main proof statistics for all methods. The last two columns are not metrics of the proof, but they indicate the total lines of code (LoC) and the total lines of specifications (LoSpec).

| Rules | Branches | Interactive steps | Quant.ins | Contract | LoopInv | LoC | LoSpec |
|---|---|---|---|---|---|---|---|
| 375,839 | 2,477 | 9,609 | 2,322 | 79 | 12 | 440 | 756 |

We found the most difficult proofs were for the method contracts of: `clear()`, `linkBefore(Object,Node)`, `unlink(Node)`, `node(int)` and `remove(Object)`. The number of interactive steps seem a rough measure for effort required. But, we note that it is not a reliable representation of the difficulty of a proof: an experienced user can produce a proof with very few interactive steps, while an inexperienced user may take many more steps. The proofs we have produced are by no means minimal.

## 4 Discussion

In this section we discuss some of the main challenges of verifying the real-world Java implementation of a `LinkedList`, as opposed to the analysis of an idealized mathematical linked list.

*Extensive use of Java language constructs.* The `LinkedList` class uses a wide range of Java language features. This includes nested classes (both static and non-static), inheritance, polymorphism, generics, exception handling, object creation and foreach loops. To load and reason about the real-world `LinkedList` source code requires an analysis tool with high coverage of the Java language, including support for the aforementioned language features.

*Support for intricate Java semantics.* The Java `List` interface is position based, and associates with each item in the list an index of Java's `int` type. The bugs described in Section 2.1 were triggered on large lists, in which integer overflows occurred. Thus, while an idealized mathematical integer semantics is much simpler for reasoning, it could not be used to analyze the bugs we encountered! It is therefore critical that the analysis tool faithfully supports Java's semantics, including Java's integer (overflow) behavior.

*Collections have a huge state space.* A Java collection is an object that contains other objects (of a reference type). Collections can typically grow to an arbitrary (but in practice, bounded) size. By their very nature, collections thus intrinsically have a large state. To make this more concrete: triggering the bugs in `LinkedList` requires at least $2^{31}$ elements (and 64 GiB of memory), and each element, since it is of a reference type, has at least $2^{32}$ values. This poses serious problems to fully automated analysis methods that explore the state space.

*Interface specifications.* Several of the `LinkedList` methods contain an interface type as parameter. For example, the `addAll` method takes two arguments, the second one is of the `Collection` type:

```
public boolean addAll(int index, Collection c) {
        ...
        Object[] a = c.toArray();
        ...
}
```

As KeY follows the design by contract paradigm, verification of `LinkedList`'s `addAll` method requires a contract for each of the other methods called, including the `toArray` method in the `Collection` *interface*. How can we specify interface methods, such as `Collection.toArray`? The stub generator generates a conservative contract: it may arbitrarily modify the heap and return *any* array. Simple conditions on parameters or the return value are easily expressed, but meaningful contracts that relates the behavior of the method to the contents of the collection require some notion of state to capture all mutations of the collection, so that previous calls to methods in the interface that contributed to the current contents of the collection are taken into account. Model fields/methods [3, Section 9.2] are a widely used mechanism for abstract specification. A model field or method is represented in a concrete class in terms of the concrete state given by its fields. In this case, as only the interface type `Collection` is known rather than a concrete class, such a representation cannot be defined. Thus the behavior of the interface cannot be fully captured by specifications in terms of model fields/variables, including for methods such as `Collection.toArray`. Ghost variables cannot be used either, since ghost variables are updated by adding set statements in method bodies, and interfaces do not contain method bodies. This raises the question: how to specify behavior of interface methods?[7]

*Verifiable code revisions.* We fixed the `LinkedList` class by explicitly bounding its maximum size to `Integer.MAX_VALUE` elements, but other solutions are possible. Rather than using integers indices for elements, one could change to an index of type `long` or BigInteger. Such a code revision is however incompatible with the general `Collection` and `List` interfaces (whose method signatures mandate the use of integer indices), thereby breaking all existing client code that uses `LinkedList`. Clearly this is not an option in a widely used language like Java, or any language that aims to be backwards compatible.

It raises the challenge: can we find code revisions that are compatible with existing interfaces and client classes? We can take this challenge even further: can we use our workflow to find such compatible code revisions, *and are also amenable to formal verification?* The existing code in general is not designed for verification. For example, the `LinkedList` class exposes several implementation details to classes in the java.util package: i.e., all fields, including `size`, are package private (not private!), which means they can be assigned a new value directly (without calling any methods) by other classes in that package. This includes setting `size` to negative values. As we have seen, the class malfunctions for negative `size` values. In short, this means that the `LinkedList` itself cannot enforce its own invariants anymore: its correctness now depends on the correctness of other classes in the package. The possibility to avoid calling methods to access the lists field may yield a small performance gain, but it precludes a modular analysis: to assess the correctness of `LinkedList` one must now analyze all classes in the same package (!) to determine whether they make benign changes (if any) to the fields of the list. Hence, we recommend to encapsulate such implementation details, including making at least all fields `private`.

*Proof reuse.* Section 3.2 discussed the proof effort (in person months). It revealed that while the total effort was 6-7 person months, once the specifications are in place after many iterations of the workflow, producing the actual final proofs took only 1-2 weeks! But minor specification changes often require to redo nearly the whole proof, which causes much delay in finding the right specification. Other program verification case studies [3,4,8,9] show similarly that the main bottleneck today is specification, not verification. This calls for techniques to optimize proof reuse when the specification is slightly modified, allowing for a more rapid development of specifications.

[7] Since the representation of classes that implement the interface is unknown in the interface itself, a particularly challenging aspect here is: how to specify the footprint of an interface method, i.e.: what part of the heap can be modified by the method in the implementing class?

*Status of the challenges.* Most of these challenges are still open. The challenge concerning "Interface specifications" could perhaps be addressed by defining an abstract state of an interface by using/developing some form of a trace specification that map a sequence of calls to the interface methods to a value, together with a logic to reason about such trace specifications.

The challenges related to code revisions and proof reuse are compounded for analysis tools that use very fine-grained proof representations. For example, proofs in KeY consist of actual rule applications (rather than higher level macro/strategy applications), and proof rule applications explicitly refer to the indices of the (sub) formulas the rule is applied to. This results in a fragile proof format, where small changes to the specifications or source code (such as a code refactoring) break the proof.

The KeY system covered the Java language features sufficiently to load and statically verify the `LinkedList` source code. KeY also supports various integer semantics, allowing us to analyze LinkedList with the actual Java integer overflow semantics. As KeY is a theorem prover (based on deductive verification), it does not explore the state space of the class under consideration, thus solving the problem of the huge state space of Java collections. We could not find any other tools that solved these challenges, so we decided at that point to use KeY.

However, other state-of-the-art systems such as Coq, Isabelle and PVS support proof *scripts*. Those proofs are described at a typically much more coarse-grained level when compared to KeY. It would be interesting to see to what extent Java language features and semantics can be handled in (extensions of) such higher level proof script languages.

### 4.1 Related work

Knüppel et al. [14] provide a report on the specification and verification of some methods of the classes ArrayList, Arrays, and Math of the OpenJDK Collections framework using KeY. Their report is mainly meant as a "stepping stone towards a case study for future research." To the best of our knowledge, no formal specification and verification of the actual Java implementation of a linked list has been investigated. In general, the data structure of a linked list has been studied mainly in terms of pseudo code of an idealized mathematical abstraction (see [18] for an Eiffel version and [12] for a Dafny version).

This paper (and [14]) has shown that the specification and verification of actual library software poses a number of serious challenges to formal verification. In our case study, we used KeY to verify Java's linked list. Other formalizations of Java also exists, such as Bali [17] and Jinja [13] (using the general-purpose theorem prover Isabelle/HOL), OpenJML [6] (a prover dedicated to Java programs), and VerCors [5] (focusing on concurrent Java programs, translated into Viper/Z3). However, these formalizations do not have a complete enough Java semantics to be able to analyze the bugs presented in this paper. In particular, these formalizations seem to have no built-in support for integer overflow arithmetic, although it can be added manually.

## Self-references

1. Bian, J., Hiep, H.A.: Verifying OpenJDK's LinkedList using KeY: Video (2019). https://doi.org/10.6084/m9.figshare.10033094.v2
2. Hiep, H.A., Maathuis, O., Bian, J., de Boer, F.S., van Eekelen, M., de Gouw, S.: Verifying OpenJDK's LinkedList using KeY: Proof Files (2019). https://doi.org/10.5281/zenodo.3517081
